# Drivers of Anuran Assemblage Structure in a Subtropical Montane Region

**DOI:** 10.1002/ece3.70624

**Published:** 2024-11-28

**Authors:** Kauan Bassetto, Vítor Carvalho‐Rocha, Carlos A. Peres, Selvino Neckel‐Oliveira

**Affiliations:** ^1^ Programa de Pós‐Graduação Em Ecologia, Departamento de Ecologia e Zoologia Universidade Federal de Santa Catarina Florianópolis Brazil; ^2^ School of Environmental Sciences University of East Anglia Norwich UK; ^3^ Instituto Jurua Manaus Brazil

**Keywords:** Atlantic Forest, elevation, elevational gradient, frogs, montane forest, species diversity

## Abstract

Elevation gradients provide excellent semi‐experimental conditions to investigate how the spatial distribution of biodiversity is shaped by the environment. Here, we investigate how temperature, productivity, and elevation are related to the diversity of anuran assemblages in a montane region of the southern Brazilian Atlantic Forest. We sampled 20 half‐hectare plots between 2020 and 2021, distributed along a sharp elevation gradient. Anuran species richness and abundance decreased with increasing elevation. We show a positive relationship between ambient temperature and frog species richness and abundance, highlighting the importance of temperature in shaping assemblages along the elevation gradient. In contrast, productivity did not affect species richness and abundance, suggesting that available energy via resources does not limit local frog diversity. We further observed marked differences in the composition of anuran assemblages between low and high elevation areas, which were related to temperature. Beta diversity is mainly driven by species replacements among assemblages, which were likely related to environmental conditions. These findings highlight the importance of incorporating protected areas that encompass the entire range of elevations to capture the full complement of landscape‐scale diversity. This is crucial as species showed limited distributions, and the marked effects of temperature should be explicitly considered under future scenarios of elevated upslope warming.

## Graphical abstract

Montane regions are known for high biodiversity and environmental conditions. Here, we investigated how these conditions influence frog diversity. Specifically, we tested whether frog richness, abundance, and composition are related to temperature and productivity between lowlands and highlands.

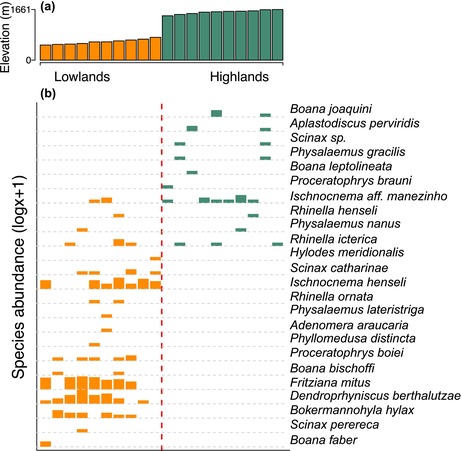

## 1 Introduction

Understanding the relationships between environmental abiotic conditions and the distribution of biodiversity across multiple scales is one of the main goals in ecology (Ricklefs 2004). Environmental gradients can provide valuable insights on how biodiversity may covary with the changes in abiotic features of the environment (García‐Navas et al. 2020). Montane regions often present sharp environmental gradients over relatively short spatial distances, which can elucidate the drivers of the distribution and abundance of local species assemblages (Haslett 1997). Thus, elevational gradients are compelling study systems due to the intrinsic diversity of montane gradients around the globe, particularly in the tropics, facilitating comparative studies (McCain 2007; Graham et al. 2014).

While the relationship between species diversity and elevation is relatively well studied (e.g., Grytnes, Heegaard, and Ihlen 2006; Villacampa et al. 2019; Carvalho‐Rocha, Peres, and Neckel‐Oliveira 2021), the mechanisms behind the drivers of community structure are still elusive (McCain and Grytnes 2010; Jarzyna, Quintero, and Jetz 2021). Montane regions account for 25% of the Earth's land surface and host a treasure trove of biodiversity, including a disproportionate number of endemic and threatened species (Rahbek et al. 2019; Karger et al. 2021). Such regions are home to about 87% of all terrestrial vertebrate diversity on Earth (Rahbek et al. 2019). Yet these environments remain undersampled, especially at very high elevations (Hughes et al. 2021), hindering assessments of changes in the distribution patterns of many taxonomic groups in the face of environmental change (Martinelli 2007).

Variation in species diversity along elevational gradients is mainly related to four major, but not mutually exclusive, drivers: Space, historical–evolutionary, climatic, and biotic processes (McCain 2009; McCain and Grytnes 2010). Habitat amount drivers, including the species–area relationship, positively associate large montane regions with higher diversity (Rosenzweig 1995). On the other hand, although montane habitat area tends to decrease with increasing elevation, this is not a general rule, with some mountains showing the largest available area at intermediate elevations (McCain 2007). Furthermore, spatial constraints alone are seldomly supported as the main factor explaining the relationships between biotic diversity and elevation, which are often related to climatic factors that are specific to the thermal optima of different taxa (McCain and Grytnes 2010).

Among climatic conditions, temperature gradually decreases at increasingly higher elevations, generally exerting a strong effect on species richness (Kessler et al. 2011) and community composition (beta diversity; Whittaker 1960) at different elevations, particularly in heterotherms (Perillo et al. 2021). In addition, temperature can limit species distributions through physiological and thermal tolerance thresholds, directly influencing local diversity (McCain and Grytnes 2010). Temperature can also affect diversity via metabolic responses and the amount of energy required to sustain different lifestyles (the metabolic‐niche hypothesis; Clarke and Gaston 2006). Furthermore, environmentally available energy is positively correlated with species diversity, and temperature is linked with both resource availability and productivity (the diversity‐energy hypothesis; Lennon, Greenwood, and Turner 2000). Primary productivity, which generally decreases at higher elevations, is another factor related to responses to elevation shifts (Sundqvist, Sanders, and Wardle 2013) and could provide an estimation for the available energy (Evans, Warren, and Gaston 2005). Even so, species richness can increase monotonically with increasing energy, according to different taxa and spatial scales (Evans, Warren, and Gaston 2005). One of the explanations of this relationship indicates that higher productivity areas can support higher population densities through greater availability of resources, at the very least maintaining species richness by precluding local extinctions (the more‐individual hypothesis; Srivastava and Lawton 1998).

These multiple mechanisms can operate simultaneously, generally resulting in four main patterns of species diversity: Decreasing, low‐elevation plateau, a low‐elevation plateau with a mid‐peak, and mid‐peak (McCain 2009). These patterns, however, may also vary according to the focal taxonomic group. For example, the diversity of tropical bats (Bogoni et al. 2021) and plants (Grytnes, Heegaard, and Ihlen 2006; Kessler et al. 2011) is generally depressed at increasingly higher elevations. On the other hand, frogs can display all four diversity patterns in roughly the same frequency (McCain and Grytnes 2010). Beta diversity patterns along altitudinal gradients, however, are relatively far less explored (but see da Silva et al. 2018, Wang et al. 2022). By decomposing beta diversity into turnover (species replacement between communities) and nestedness (species gain or loss among communities) components (Baselga 2010), we can improve our understanding of the processes behind compositional changes (Anderson et al. 2011; Soininen, Heino, and Wang 2018). Turnover patterns can be associated with environmental dispersal and filtering processes, while nestedness can usually occur as a result of colonization and extinction processes (da Silva et al. 2018; Soininen, Heino, and Wang 2018). In this context, since different climatic conditions are found along elevation gradients, turnover patterns are expected due to species specificity to different conditions (Jankowski et al. 2009).

Despite the increase in the number of studies seeking to understand the relationships between species diversity and elevation in the last decades, taxonomic groups like amphibians are still underrepresented. This is particularly true over the extensive elevation gradients along subtropical regions, which remain poorly sampled (Carvalho‐Rocha, Peres, and Neckel‐Oliveira 2021). Amphibians represent a remarkably diverse vertebrate group, comprising approximately 8707 species worldwide (Frost 2024), 1188 of which are found in Brazil (Segalla et al. 2021). This group stands out as the most endangered among all vertebrates, facing multiple threats, especially due to habitat loss, climate change, and infectious diseases (Luedtke et al. 2023). The Brazilian Atlantic Forest harbors 625 amphibian species (Rossa‐Feres et al. 2017) and stands out given its marked concentration of endangered taxa (Luedtke et al. 2023). However, this biome has succumbed to high historical deforestation and habitat fragmentation, with only < 16% of its original forest cover remaining, underscoring concerns over this global amphibian hotspot (Ribeiro et al. 2009). Yet, studies on amphibian diversity in montane environments in this biome are still scarce, especially in the southernmost subtropical regions, with a major focus on species inventories (Campos and Lourenço‐de‐Moraes 2017). Carvalho‐Rocha, Peres, and Neckel‐Oliveira (2021) carried out the only study to date in such montane environments and focused on lentic reproductive sites, such as puddles and ponds, where only a fraction of the regional gamma diversity can be sampled (and at that mainly breeding males). Sampling restricted to water bodies, therefore, also excludes other environments and species with alternative reproductive modes, including terrestrial breeding species that lack a larval phase.

Since temperature and productivity can influence amphibian diversity, here we seek to test the hypothesis that high‐elevation regions, over 800 m above sea level (masl), that are subjected to lower temperatures and less productivity host a lower anuran diversity in relation to adjacent lowlands. We then assess the structure of terrestrial anuran communities, including species richness, overall abundance, and composition, along different elevations to answer the following questions: (1) Is anuran species richness and abundance positively related to temperature? We expect that high elevations host lower richness and abundance, given that temperature can affect diversity through physiological tolerance constraints, resource availability, and metabolic rates; (2) Is anuran species richness and abundance positively related to productivity? We expect that low elevations host higher richness and abundance, given that they contain higher productivity and consequently more available resources; (3) Does the composition of anuran assemblages change between low and high elevations, and if so, to what degree is this accounted for by temperature and/or productivity? We expect that anuran composition will vary with elevation due to differences in associated environmental conditions; and finally (4) Are dissimilarities among anuran assemblages due to replacement or gain/loss of species and individuals? We expect that species turnover will account for the dominant fraction of total beta diversity along the elevational gradient due to associated changes in environmental conditions.

## 2 Methods

### 2.1 Study Area

**FIGURE 1:**
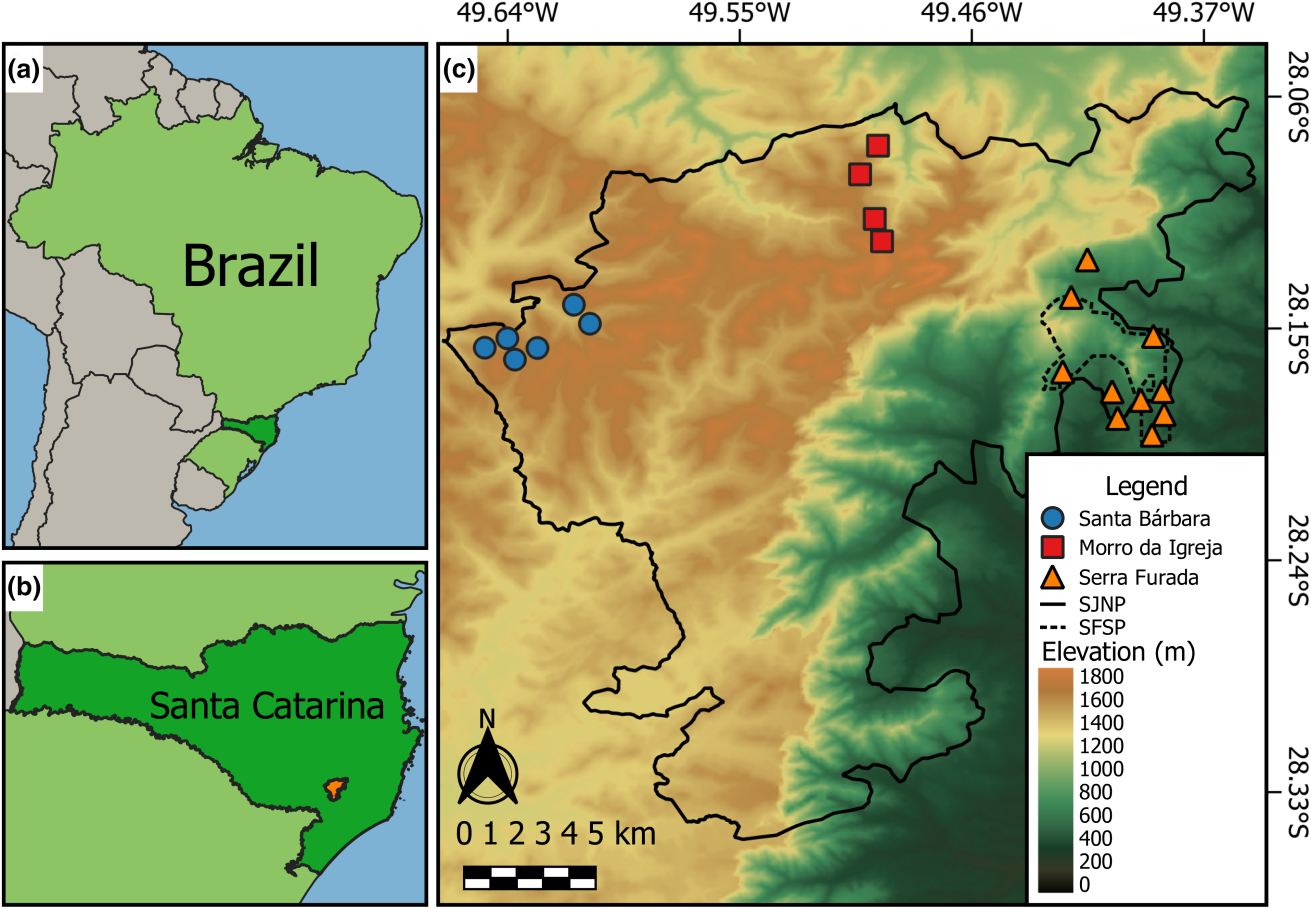
Geographic location of the study area. (a) Map of Brazil, highlighting the southern state of Santa Catarina; (b) Map of Santa Catarina highlighting in orange the São Joaquim National Park (SJNP) and the Serra Furada State Park (SFSP); (c) Topographic map of SJNP and SFSP, indicating the sample plots distributed across three clusters: Santa Bárbara, Morro da Igreja, and Serra Furada indicated by blue circles, red squares, and orange triangles, respectively.

Approximately 12% of the Atlantic Forest biome is represented by montane environments (Bicudo da Silva et al. 2020), with the southern state of Santa Catarina hosting one of the largest remaining areas of the original vegetation cover of the Atlantic Forest within Brazil (Ribeiro et al. 2009). In this context, our study was carried out within two protected areas in Santa Catarina, southern Brazil: The São Joaquim National Park (SJNP) and the Serra Furada State Park (SFSP) (Figure 1). These protected areas harbor high environmental heterogeneity given their wide variation in elevation, ranging from 300 to 1822 masl (de Novaes Vianna et al. 2015). The vegetation up to 800 masl (lowlands) is predominantly composed of dense ombrophilous forest in advanced successional stages, while areas above 800 masl (highlands) are composed of mixed ombrophilous *Araucaria* forest interspersed with natural grasslands and cloud forests (IBGE 2012). According to the Köppen classification, the climate is humid subtropical lacking a marked dry season, where areas below 700 masl are humid mesothermal (Cfa) with a hot summer, and those above 700 masl experience a temperate summer (Cfb) (Alvares et al. 2013). The combination of high subtropical latitudes and high elevations results in one of the most climatically contrasting montane environments across the Neotropics, with upland areas above 700 masl frequently experiencing multiple cold fronts and hard frosts during winter, reaching down to −8°C (Nimer 1989).

### 2.2 Data Collection

#### 2.2.1 Anuran Sampling

We sampled anurans at 20 plots, including 10 plots located between 300 and 800 masl (lowlands) and 10 between 800 and 1820 masl (highlands; Table S1). Our 5000‐m^2^ plots followed the spatial configuration of the RAPELD biodiversity inventory network (Magnusson et al. 2005) and consisted of a 20 × 250 m area, spaced apart by a minimum distance of 1 km (Figure 1c). Given the topographic heterogeneity of our study area, plots were placed at three subregions: Santa Bárbara, *N* = 6; Morro da Igreja, *N* = 4; and Serra Furada, *N* = 10 (see Figure 1c).

Sampling at each plot was conducted during the reproductive season of anurans over two consecutive years (2020 and 2021), which spans from mid‐summer to early autumn, between January and March. In each sampling campaign, two experienced observers systematically conducted 1 h surveys, once during daylight and once during the night period, resulting in a total sampling effort of 4 person‐hours per plot. We awaited at least 1 h between diurnal and nocturnal sampling periods to avoid any influence between them. We employed a combination of visual and acoustic search methods to intensively search for frogs throughout each 5000‐m^2^ plot (Rödel and Ernst 2004). Additionally, for auditory records, we only registered a new individual that was near to researchers as long as they advanced to the plot end. For visual records, individuals were captured for species identification and then released behind the direction of the survey. The species taxonomy and nomenclature used in this study have been updated according to Frost (2024).

#### 2.2.2 Environmental Variables

We used the geographic centroid of each surveyed plot to extract its elevation (ELE) from the TOPODATA digital elevation model (30 m pixel resolution; de Morisson Valeriano and de Fátima Rossetti 2012). We also extracted values of Mean Annual Temperature (MAT), which were obtained from the WorldClim database (1 km pixel resolution; Fick and Hijmans 2017). Additionally, we used Net Primary Production (NPP) as a proxy of productivity, extracted from the MOD17A3HGFv061 product of Terra MODIS database (at 500 m pixel resolution; Running and Zhao 2021). For this, we created a 500 m buffer around plot centroids and calculated the mean of NPP pixel values within each buffer area for 2020 and 2021 and calculated the mean between years. These steps were performed in Google Earth Engine (Gorelick et al. 2017). The MAT and ELE variables were extracted using the QGIS software with the Albers Equal Area Conic projection (QGIS Development Team 2023).

### 2.3 Data Analysis

For analytical purposes, we considered the maximum number of individuals recorded for each frog species across both survey periods (i.e., day and night) and years at each plot as a measure of local abundance. We used this approach to avoid double‐counting the same individual, which could have overestimated the total frog abundance (Dias‐Terceiro et al. 2015). As species richness regard, we use the total number of species sampled in each plot during the 2 years of sampling. We evaluated sample effort completeness using coverage‐based methods with the “*iNEXT”* R package (Hsieh, Ma, and Chao 2016). Given the high variation in species richness across sites, we applied the “*estimateD*” function to further assess sample effort completeness. To standardize comparisons, we used the minimum estimated coverage to compare with observed richness values. Finally, we performed a Spearman correlation to examine the relationship between estimated and observed values.

We then used generalized linear models (GLMs) to investigate the relationship between frog species richness and abundance with elevation, temperature, and productivity. We used a negative binomial error distribution for species richness and local abundance to account for overdispersion of the data. For each of our response variables (i.e., species richness and abundance), we fitted separated models using two sets of predictors: (1) elevation only and (2) mean annual temperature and net primary productivity. We used this approach to, first, evaluate the overall diversity pattern along the elevational gradient and then evaluate the importance of mean annual temperature and net primary productivity in explaining the spatial patterns depicted in the first model. We only included in the models predictors that had a variance inflation factor (VIF) lower than 5 (Zuur et al. 2009), calculated using the “*car”* R package (Fox et al. 2012). Finally, we further used the “*DHARMa*” R package to perform model validation (Hartig and Hartig 2017).

We assessed differences in frog species composition between sites at low and high elevation levels using a multivariate generalized linear model (GLMmv) with the package “*mvabund*” (Wang et al. 2012). We used the abundance data with a negative binomial error distribution and elevation (i.e., either lowlands or highlands) as a categorical variable. Moreover, we performed GLMmv's with the other environmental variables (MAT and NPP) to investigate their relationships with frog species composition (negative binomial error distribution). In addition, we also calculated the Akaike information criteria of models to compare and identify the best in explaining frog compositional variation. Finally, we assessed the variation explained by predictor variables using pseudo‐*R*‐squared (following Slavich et al. 2014). To visualize differences in frog species composition between elevations (i.e., lowlands and highlands), we used a principal coordinate analysis (PCoA) based on the Bray–Curtis dissimilarity matrix estimated from the abundance data. For all GLM and GLMmv models with more than one predictor variable, we applied the “*scale*” function from the R base package to standardize the variables.

We calculated beta diversity among sites using pairwise Sørensen (incidence‐based) and Bray‐Curtis (abundance‐based) dissimilarity indices. Sørensen dissimilarity (*β*_SOR_) represents the total beta diversity and can be decomposed in two components representing antithetic processes: Turnover and nestedness components (i.e., species replacement (*β*_SIM_) and species gain/loss (*β*_SNE_), respectively; Baselga 2010). Similarly, Bray–Curtis dissimilarity (*β*_BC_) can be partitioned in two components: Balanced variation in abundance and abundance gradient (i.e., substitution of a number of individuals in one site by the same number of individuals of different species in another site (*β*_BC.BAL_) and abundance gain/loss (*β*_BC.GRA_), respectively; Baselga 2017). For each index and their components, we generated a multisite‐pairwise matrix with the “*beta.pair*” and “*beta.pair.abun*” functions of “*betapart*” package (Baselga and Orme 2012). Further, we accessed the environmental dissimilarities (elevation, temperature, and productivity distances) for each pairwise site using Euclidean distance. To investigate the relationship between beta diversity and its components with each environmental predictor, we perform individual GLMs with gama error distribution for each predictor with *“betareg”* function of “*betareg”* package (Cribari‐Neto and Zeileis 2010). All statistical analyses were performed under the R environment, version 4.1.0 (R Core Team 2021), and all data are available in the Data S1 (Bassetto et al. 2024).

## 3 Results

**FIGURE 2:**
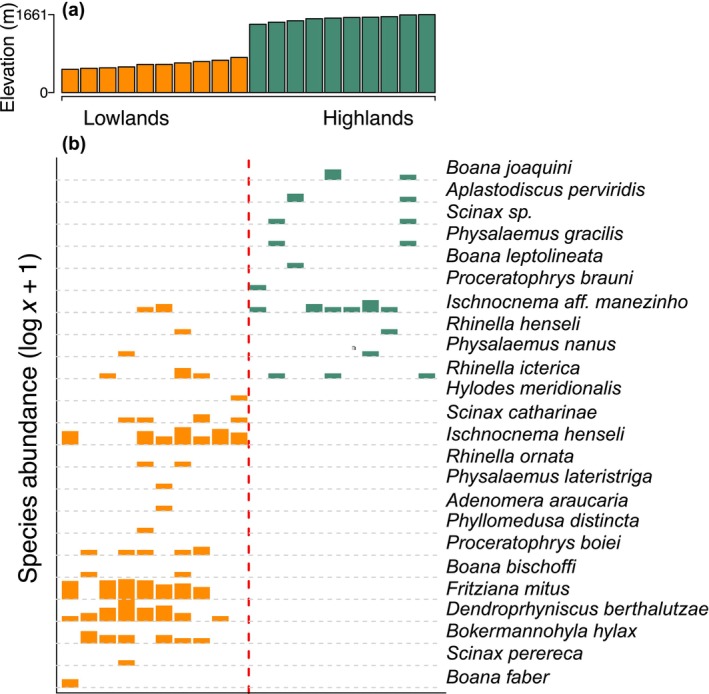
Species occurrence and abundance profile across low‐elevation (orange bars) and high‐elevation plots (green bars) in our study area within the São Joaquim National Park and Serra Furada State Park. (a) Plots are ordered left to right from low‐ to high‐elevation (in meters above sea level). (b) Variation in total abundance (log *x* + 1) per species found at each plot, scaled by the height of vertical bars.

In total, we recorded 208 individuals representing 24 species and 7 families of Neotropical anurans (Table S2), with a mean of 10 individuals (range = 1–36) and 3 species (range = 1–9) per plot. There was a marked difference in the overall anuran abundance across elevations. While 180 (86.5%) individuals were sampled at low‐elevation plots, only 28 (13.4%) were sampled at high‐elevation plots. Species richness followed the same pattern, with 18 (75%) species recorded at low‐elevation plots but only 10 (41.6%) at high‐elevation plots (Figure 2). The most species‐rich (9 species) and maximum abundance (36 individuals) were also recorded at low‐elevation plots. The observed and estimated frog species richness (based on minimum sample coverage) per plot showed a high positive correlation (rho = 0.65; *S* = 475.47; *p*‐value = 0.001; Figure S1). Over half of all species (14 species, 58%) were unique to low‐elevation plots, with *Fritizana mitus* and *Dendrophryniscus berthalutzae* being the most abundant (*N* = 62 and *N* = 40, respectively; Figure 2; Table S2). On the other hand, six species (25%) were only recorded at the high‐elevation plots, with *Boana joaquini* as the most prevalent species (*N* = 4). Only 4 out of 24 species (16.7%) were found at both low‐ and high‐elevation plots, of which *Ischnocnnema* aff. *manezinho* was the most abundant (*N* = 13; Figure 2), highlighting a marked overall turnover between elevation zones.

**FIGURE 3:**
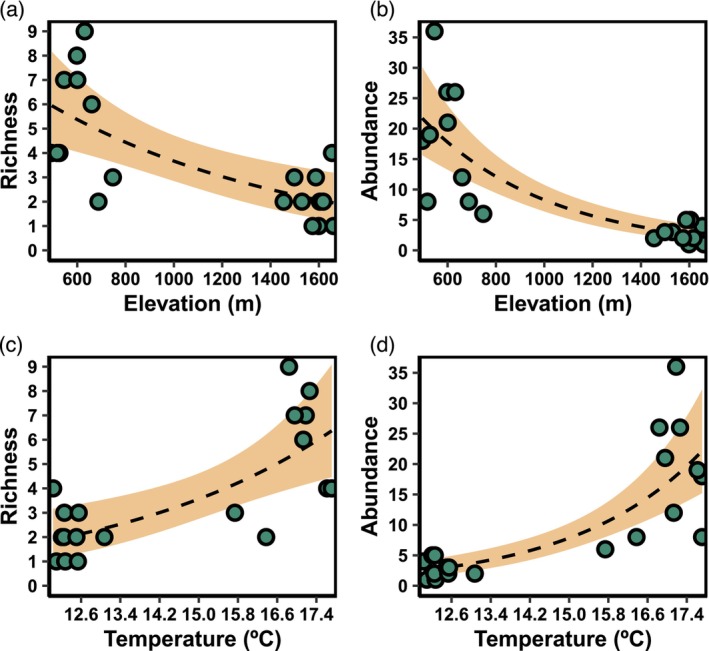
Predictions from Generalized Linear Models. (a) and (b) show the relationships between elevation and anuran species richness and abundance, respectively. (c) and (d) show the relationships between mean annual temperature and anuran species richness and abundance, respectively. Green circles, dashed lines, and shaded areas represent observed values, nonlinear model predictions, and 95% confidence intervals, respectively.

Frog species richness showed a clear decreasing pattern with increasing plot elevation (*R* ^2^ = 0.50, estimate = −0.0009, *p* < 0.001; Figure 3a). Conversely, we found the opposite pattern for temperature and richness (*R* ^2^ = 0.50, estimate = 0.5232, *p* < 0.001), where warmer sites showed higher species richness (Figure 3c). We found no evidence that variation in productivity could be related to species richness (Table S3). Similar to species richness, frog abundance also declined with increasing elevation (*R* ^2^ = 0.47, estimate = −0.0019, *p* < 0.001; Figure 3b). In contrast, temperature was positively related to abundance (*R* ^2^ = 0.40, estimate = 0.936, *p* < 0.001; Figure 3d), indicating higher abundance at warmer sites. We also found no evidence that frog abundance could be related to variation in productivity (Table S3).

**FIGURE 4:**
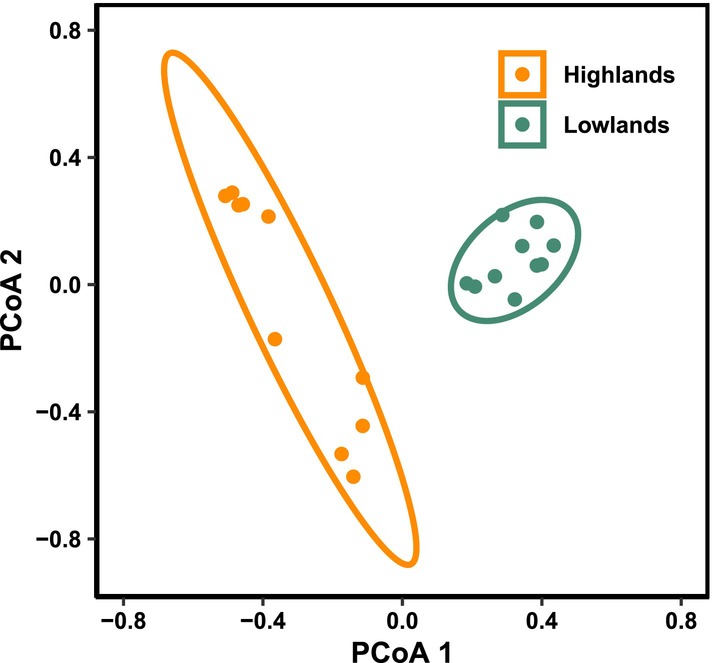
Compositional differences between anuran assemblages at two major elevational zones. First two axes of a Principal Coordinate Analysis (PCoA), based on Bray–Curtis dissimilarity, showing the clear divergence between low‐elevation (green) and high‐elevation (orange) anuran assemblages.

There were marked compositional differences between low‐ and high‐elevation plots (LR = 108.7, *p* = 0.001, Pseudo‐*R* ^2^ = 0.29; Figure 4; Table S4), with a total of 18 out of 24 frog species occurring in low‐elevation sites. Concerning the environmental variables, the best model included only the mean annual temperature (LR = 111.9, *p* = 0.001; Pseudo‐*R* ^2^ = 0.30), suggesting it as a key predictor in exacerbating these compositional differences (Table S5). Such differences were also evident in terms of species composition from the principal coordinate analysis (axis 1 = 32.20% and axis 2 = 19.22%), clearly showing that low‐elevation assemblages diverged from those at high‐elevation (Figure 4).

**FIGURE 5:**
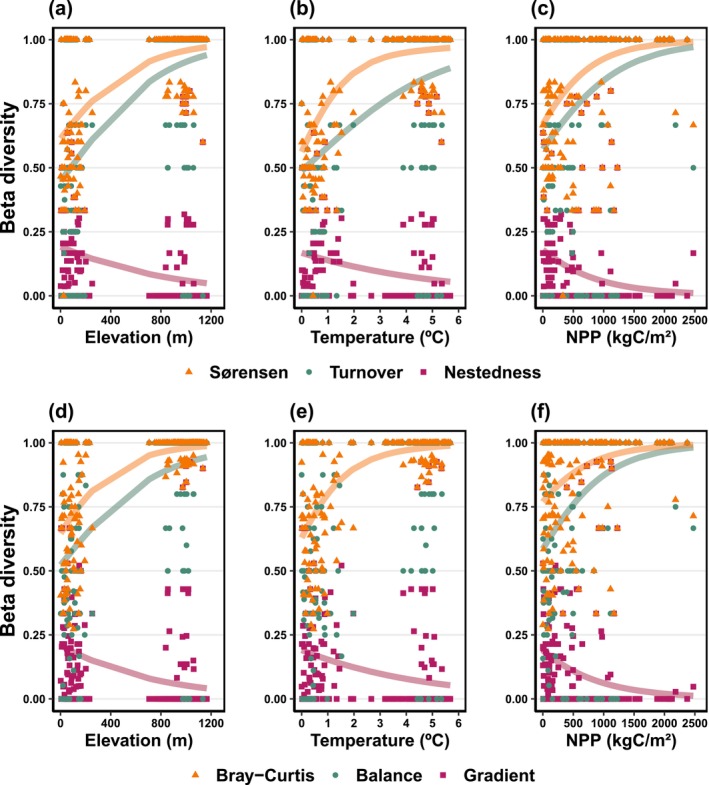
Relationships between total beta diversity and its components with different sources of environmental distance. Total beta diversity measured using both incidence‐based (Sørensen) and abundance‐based indices (Bray–Curtis) are represented by orange triangles; turnover and balanced abundance components are indicated by green triangles, while nestedness and abundance gradient are represented by purple squares. Geometric dots represent observed dissimilarities, and lines represent linear model predictions for significant relationships (*p* < 0.5).

We also observed an overall increase in dissimilarity between anuran assemblages with increasing elevational distances for both the presence–absence (Sørensen: estimate = 0.0013, SE = 0.0001, *p*‐value < 0.001, Pseudo‐*R* ^2^ = 0.41; Figure 5a) and the abundance‐based data (Bray–Curtis: estimate = 0.0017, SE = 0.0001, *p*‐value < 0.001, Pseudo‐*R* ^2^ = 0.61; Figure 5d). Disentangling the beta diversity components, the turnover (estimate = 0.0016, SE = 0.0002, *p*‐value < 0.001, Pseudo‐*R* ^2^ =  0.06; Figure 5a) and balanced variation in abundance (estimate = 0.0016, SE = 0.0002. *p*‐value < 0.001, Pseudo‐*R* ^2^ = 0.18; Figure 5d) showed a positive relationship with the elevational distance between sites. In contrast, both nestedness (estimate = −0.0007, SE = 0.0001, *p*‐value < 0.001, Pseudo‐*R* ^2^ = 0.06; Figure 5a) and abundance gradient (estimate = −0.0013, SE = 0.0002, *p*‐value < 0.001, Pseudo‐*R* ^2^ = 0.10; Figure 5d) showed a negative relationship with the elevational distance between sites. Similarly, overall dissimilarity between anuran assemblages was increasing with temperature distance for both beta diversity indices, Sørensen (estimate = 0.1174, SE = 0.0216, *p*‐value < 0.001, Pseudo‐*R* ^2^ = 0.60; Figure 5b) and Bray–Curtis (estimate = 0.2846, SE = 0.0314, *p*‐value < 0.001, *R* ^2^ = 0.59; Figure 5e). For beta diversity components, turnover (estimate = 0.0181, SE = 0.0078, *p*‐value = 0.02, Pseudo‐*R* ^2^ = 0.23; Figure 5b) showed a positive relationship with temperature distance, while nestedness (estimate = −0.1615, SE = 0.0719, *p*‐value = 0.02, Pseudo‐*R* ^2^ = 0.06; Figure 5b) and the abundance gradient (estimate = −0.2153, SE = 0.0677, *p*‐value = 0.001, Pseudo‐*R* ^2^ = 0.08; Figure 5e) showed negative relationships. The balanced variation in abundance showed no relationship with temperature (Table S6). Finally, the total beta diversity of anuran was positively related with productivity distance for incidence‐ and abundance‐based indices (Sørensen: estimate = 0.0012, SE = 0.0002, *p*‐value < 0.001, Pseudo‐*R* ^2^ = 0 and Bray–Curtis: estimate = 0.0009, SE = 0.0001, *p*‐value < 0.001, Pseudo‐*R* ^2^ = 0.14; Figure 5c,f, respectively). The turnover and balanced variation in abundance showed a positive relationship with productivity distance (estimate = 0.0008, SE = 0.0002, *p*‐value < 0.001, Pseudo‐*R* ^2^ = 0, Figure 5c and estimate = 0.0011, SE = 0.0002, *p*‐value < 0.001, Pseudo‐*R* ^2^ = 0, Figure 5f, respectively). Moreover, nestedness (estimate = −0.0008, SE = 0.0001, *p*‐value < 0.001, Pseudo‐*R* ^2^ = 0.04; Figure 5b) and abundance gradient (estimate = −0.0008, SE = 0.0001, *p*‐value < 0.001, Pseudo‐*R* ^2^ = 0.04; Figure 5e) followed the same pattern as elevation, decreasing with the productivity distance. Otherwise, these results indicate that both incidence‐based and abundance‐based beta diversity are driven by species’ and individuals’ turnover in local assemblages across environmental distances.

## 4 Discussion

In this study, we investigated the link between variation in the environmental setting and the landscape‐scale diversity and composition of anuran species in a subtropical montane region. The expected variation in pond‐breeding anuran communities is attributed to differences in elevation and microclimates (Carvalho‐Rocha, Peres, and Neckel‐Oliveira 2021). Our results are consistent with the notion that temperature acts as an important driver shaping the wider anuran assemblages, including terrestrial breeding species. Productivity, however, was unrelated to variation in anuran species diversity and composition in this subtropical montane region.

We found a clear reduction in anuran species richness with increasing elevation, which is consistent with other anuran studies in tropical mountains (Zancolli, Steffan‐Dewenter, and Rödel 2014; Villacampa et al. 2019). Subtropical ecosystems were also shown to have more species‐rich assemblages at lowlands and intermediate elevations compared to highlands (Carvalho‐Rocha, Peres, and Neckel‐Oliveira 2021; Zhao et al. 2022). Such pattern is apparently widespread among multiple ectothermic taxa, as previously reported for reptiles (McCain 2010), ants (Longino and Colwell 2011), and bees (Classen et al. 2015). In other studies, anuran species richness and abundance are positively correlated with mean annual temperature (Carvalho‐Rocha, Peres, and Neckel‐Oliveira 2021; Matavelli et al. 2022). Variation in temperature across marked elevational gradients is one of the main drivers of continental to global scale diversity in many organisms (Peters et al. 2016), especially in ectothermic vertebrates like anurans (Carvalho‐Rocha, Peres, and Neckel‐Oliveira 2021, Matavelli et al. 2022). Their susceptibility to thermal fluctuations, coupled with limited dispersal capacity, can restrict less tolerant species from becoming established in cold microhabitats at high elevations (Zuloaga and Kerr 2017). Indeed, temperature affects anuran physiology, behavior, and ecology, influencing growth, locomotion rates, spatial distribution, and local diversity (Navas 2002). In highly seasonal subtropical areas, the influence of thermal niches extend to anuran assemblages, with cold‐intolerant species exhibiting restricted winter activity compared to cold‐tolerant counterparts (Ceron et al. 2020). The condition of inherently low seasonal temperatures at high elevations can also limit community‐wide anuran abundance (Carvalho‐Rocha, Peres, and Neckel‐Oliveira 2021), potentially leading to low species detectability and affecting local diversity estimates. Overall low population densities at high elevations may be caused by the detrimental effect of low temperatures in the reproductive success of most anuran species (Kiss et al. 2009). It is important to note that we cannot rule out additional factors in this study, such as colonization history and water availability, which could also influence anuran diversity along the elevational gradient (Zancolli, Steffan‐Dewenter, and Rödel 2014). This is particularly relevant for species restricted to specific microhabitat conditions at high elevations, as they may face heightened exposure to variable, if not extreme, temperatures. Other analytical approaches could further elucidate the processes resulting in the patterns we found, including phylogenetic, functional, and historical colonization processes (Benício et al. 2021).

In contrast, productivity was not associated with variation in anuran species richness and abundance. This is at odds with observations on amphibian species richness for tropical and subtropical areas of South America (Gouveia et al. 2013). In fact, productivity can contribute positively to abundance (Perner et al. 2005) and arthropod diversity (Schuldt et al. 2019). In tropical areas, even at short elevation distances, the variation in environmental conditions can influence the diversity of hymenopterans (Perillo et al. 2021), which contributes heavily to the diet of anuran amphibians (Solé et al. 2005; Carvalho‐Rocha, Lopes, and Neckel‐Oliveira 2018). Thus, productivity may indirectly influence the diversity of these organisms, considering that greater resource availability at more productive sites can boost the abundance of many frog and toad populations. Our results, however, failed to show a relationship between productivity and both anuran species richness and abundance. This suggests that energy availability may not be a limiting factor for amphibian diversity in our study system, as previously discussed for pond amphibians (Carvalho‐Rocha, Peres, and Neckel‐Oliveira 2021). On the other hand, the use of productivity proxy generated from remote sensing may present limitations in investigating their relationship with species diversity, particularly when compared to productivity data collected at a local scale (for further discussion, see Šímová and Storch 2017). Moreover, while model‐based productivity proxies can explain amphibian diversity at global and regional scales (e.g., Gouveia et al. 2013), direct field measurements of productivity would enhance our understanding of the amphibian–productivity relationship at smaller scales. Therefore, further studies investigating the spectrum and amount of trophic resources and their relationships with productivity and climate (e.g., temperature and precipitation) are thus necessary to directly investigate the relationships between productivity, resources, abundance, and richness (McCain et al. 2018).

Amphibians use a wide spectrum of microhabitats, which are primarily associated with the diversity of reproductive modes (Haddad and Prado 2005). In this sense, environmental features could be a decisive factor in structuring anuran assemblages (Naniwadekar and Vasudevan 2007; Carvalho‐Rocha, Peres, and Neckel‐Oliveira 2021). For instance, at low‐elevation plots, the notably high abundance of species such as *Dendrophryniscus berthalutzae* and *Fritziana mitus* was likely linked to the widespread presence of epiphytic bromeliads. These species use the water accumulated in bromeliad tanks to deposit their eggs and raise tadpoles (Izecksohn 1993; Walker et al. 2018). The generally insufficient availability of aquatic environments as breeding microhabitats for water‐dependent species is likely a key driver of the low anuran species diversity at high elevations (Villacampa et al. 2019). On the other hand, species such as *Ischnocnema* aff. *manezinho*, which directly oviposits underneath the forest leaf litter (Haddad and Prado 2005), was most abundant at high‐elevation plots. Its upland dominance may be related to the lower water availability at high elevation, as terrain slopes tend to be steeper, thereby hindering the accumulation of water into perennial ponds (Catenazzi 2011). Moreover, although we focused on two important drivers for amphibian species diversity (i.e., temperature and productivity), we emphasize the importance adopting complementary approaches to better understand montane amphibian diversity. As previously mentioned, future studies exploring the relationships between microhabitats, habitat structure, and water availability, and their effects on amphibian diversity, are needed to unravel montane assemblages (Pitogo et al. 2021).

Overall, beta diversity (Sørensen and Bray‐Curtis) and turnover (incidence and abundance) showed a positive relationship with environmental distance. For instance, we found that assemblage dissimilarity, primarily via species substitutions, intensifies with increasing environmental distances in elevation, temperature, and productivity. Our results mirror those of others’ studies showing that amphibian beta diversity is driven by turnover across elevational gradients (e.g., Wang et al. 2022, Kang et al. 2024, for larval phase and adults, respectively). Furthermore, elevation distance usually reflects the same variation in others environmental distance measurements, implying divergence in environmental characteristics along the elevation gradient. This supports the relationship between environmental characteristics and the structuring of communities in our study system, since environmental change with increasing distance and spatial separation of species with different environmental requirements is one of the mechanisms that can explain this pattern (Soininen, McDonald, and Hillebrand 2007). The fact that species composition diverged markedly between lowland and highland sites suggests environmental filters for distinct sets of species, mainly due to species that are well adapted to the most extreme high‐elevation temperatures.

Some highland species, such as *Boana joaquini* , are associated with montane regions, preferring streams near open areas and forest edges within elevations between 1245 and 1730 m asl (Garcia, Vinciprova, and Haddad 2003). Furthermore, species such as *Boana leptolineata* and *Physalaemus gracilis* are more likely to occur in cold regions (Carvalho‐Rocha, Peres, and Neckel‐Oliveira 2021). Our findings suggest that temperature plays a pivotal role in shaping anuran assemblages, implying an environmental filtering mechanism in which the occurrence of cold‐adapted species is limited at much warmer lowland sites. In this case, specialization to survive in colder environments may have resulted in a tradeoff with the ability to compete for resources (e.g., space), thereby precluding the establishment of those species at low elevations. The observed patterns of diversity along elevation gradients are the result of both ecological and evolutionary interactions (Lomolino 2001). Further information on the relationships between anuran reproductive modes and availability of water bodies at varying elevations are needed to better understand the distribution patterns of these amphibians in (sub)tropical mountains (Siqueira et al. 2021). Additionally, more evolutionary biogeography studies are necessary to explore the possible drivers of species distribution in these environments (García‐López, Micó, and Galante 2012).

To conclude, our study shows that variations in elevation and temperature are important in structuring amphibian assemblages in a montane system in subtropical South America. Furthermore, species replacements among assemblages along the altitudinal gradient highlight the conservation importance of retaining the entire geographic macromosaic for both the origin and maintenance of the regional species pool. Natural and relatively intact vegetation accounts for nearly 50% of the overall land cover in montane regions of the Atlantic Forest biome. Considering the entire biome at any elevation, this proportion drops to a quarter of the land cover, but only less than 9% of these areas are strictly protected (Bicudo da Silva et al. 2020). Thus, conservation planning should consider well‐distributed protected areas throughout the elevation gradient, as well as connectivity between elevation zones, not least because subtropical and tropical mountains are under severe threats of habitat loss and fragmentation (Karger et al. 2021). Given future scenarios of climate change, monitoring these communities has become increasingly important to understand how biodiversity will respond to rising temperatures across tropical mountains.

## Author Contributions

**Kauan Bassetto:** conceptualization (equal), data curation (lead), formal analysis (equal), writing – original draft (lead). **Vítor Carvalho‐Rocha:** conceptualization (equal), formal analysis (equal), writing – review and editing (equal). **Carlos A. Peres:** conceptualization (equal), writing – review and editing (equal). **Selvino Neckel‐Oliveira:** conceptualization (equal), funding acquisition (lead), writing – review and editing (equal).

## Conflicts of Interest

The authors declare no conflicts of interest.

## Supporting information


Data S1.

Data S2.

## Data Availability

All the data generated or analyzed during this study are provided in Data S1 (Bassetto et al. 2024).
