# Supplementary material for: Drivers of Anuran Assemblage Structure in a Subtropical Montane Region
Source: Ecol Evol. 2024 Nov 28;14(12):e70624. doi: 10.1002/ece3.70624 (PMC11602670; doi:10.1002/ece3.70624)
Supplement: Supplementary file 2 — Data S2. [file ECE3-14-e70624-s001.docx]

**Supplementary Information**

Table S1 - Plot names, geographic coordinates, elevation, and variables collected at the three clusters of sampling plots within the SJPN and SFSP region, southern Brazil.

| Site | Longitude | Latitude | Elevation (m) | MAT (ºC) | NPP (kgC/m²) | Region |
| --- | --- | --- | --- | --- | --- | --- |
| L400 | -49.4963 | -28.0791 | 1454 | 13.1 | 19449.75 | Morro da Igreja |
| PTN0400 | -49.6143 | -28.1408 | 1599 | 12.1 | 18573.125 | Santa Bárbara |
| PTS3500 | -49.6372 | -28.1621 | 1654 | 12 | 17370 | Santa Bárbara |
| T1 | -49.3854 | -28.1833 | 495 | 17.7 | 19370.25 | Serra Furada |
| T10 | -49.4247 | -28.1669 | 660 | 17.1 | 18960.125 | Serra Furada |
| T2 | -49.3944 | -28.1779 | 599 | 17.3 | 19458.25 | Serra Furada |
| T4 | -49.4034 | -28.1849 | 526 | 17.6 | 19456.875 | Serra Furada |
| T5 | -49.39 | -28.1914 | 516 | 17.7 | 19455.5 | Serra Furada |
| T6 | -49.3895 | -28.1531 | 631 | 16.8 | 19443.25 | Serra Furada |
| T7 | -49.4151 | -28.1233 | 747 | 15.7 | 19249.33333 | Serra Furada |
| T8 | -49.4055 | -28.1746 | 546 | 17.2 | 19355.625 | Serra Furada |
| W1500 | -49.5033 | -28.0902 | 1604 | 12.2 | 19492.5 | Morro da Igreja |
| W4500 | -49.4948 | -28.1162 | 1587 | 12.3 | 19551.75 | Morro da Igreja |
| PTS2500 | -49.6285 | -28.1576 | 1660 | 12.3 | 18326.125 | Santa Bárbara |
| PTN3500 | -49.6401 | -28.1539 | 1618 | 12.3 | 19743.25 | Santa Bárbara |
| PTN4500 | -49.6489 | -28.1575 | 1530 | 12.5 | 19844.625 | Santa Bárbara |
| W3500 | -49.4976 | -28.1075 | 1574 | 12.5 | 18903.25 | Morro da Igreja |
| PTS0500 | -49.6081 | -28.1482 | 1498 | 12.6 | 18482.75 | Santa Bárbara |
| T3 | -49.386 | -28.1744 | 600 | 17 | 19297.5 | Serra Furada |
| T9 | -49.4214 | -28.1379 | 688 | 16.4 | 19440.75 | Serra Furada |

Table S2 **-** Abundance of anuran families and species sampled at both low and high elevations in the region of the São Joaquim National Park and Serra Furada State Park in southern Brazil.

|  | **Abundance** | | |
| --- | --- | --- | --- |
| **Family/Species** | **Lowlands** | **Highlands** | **Total** |
| **Brachycephalidae** |  |  |  |
| *Ischnocnema henselii* (Peters, 1870) | 34 |  | 34 |
| *Ischnocnema* aff. *manezinho* (Garcia, 1996) | 3 | 10 | 13 |
| **Bufonidae** |  |  |  |
| *Dendrophryniscus berthalutzae* Izecksohn, 1994 | 40 |  | 40 |
| *Rhinella henselii* (Lutz, 1934) | 1 | 1 | 2 |
| *Rhinella icterica* (Spix, 1824) | 5 | 3 | 8 |
| *Rhinella ornata* (Spix, 1824) | 2 |  | 2 |
| **Hemiphractidae** |  |  |  |
| *Fritziana mitus* Walker *et* *al*., 2018 | 62 |  | 62 |
| **Hylidae** |  |  |  |
| *Aplastodiscus perviridis* Lutz, 1950 |  | 3 | 3 |
| *Boana bischoffi* (Boulenger, 1887) | 2 |  | 2 |
| *Boana faber* (Wied-Neuwied, 1821) | 2 |  | 2 |
| *Boana joaquini* (Lutz, 1968) |  | 4 | 4 |
| *Boana leptolineata* (Braun & Braun, 1977) |  | 1 | 1 |
| *Bokermannohyla hylax* (Heyer, 1985) | 12 |  | 12 |
| *Phyllomedusa distincta* Lutz, 1950 | 1 |  | 1 |
| *Scinax catharinae* (Boulenger, 1888) | 5 |  | 5 |
| *Scinax perereca* Pombal, Haddad & Kasahara, 1995 | 1 |  | 1 |
| *Scinax* sp. |  | 2 | 2 |
| **Hylodidae** |  |  |  |
| *Hylodes meridionalis* (Mertens, 1927) | 1 |  | 1 |
| **Leptodactylidae** |  |  |  |
| *Adenomera araucaria* Kwet & Angulo, 2002 | 1 |  | 1 |
| *Physalaemus gracilis* (Boulenger, 1883) |  | 2 | 2 |
| *Physalaemus lateristriga* (Steindachner, 1864) | 1 |  | 1 |
| *Physalaemus nanus* (Boulenger, 1888) | 1 | 1 | 2 |
| **Odontophrynidae** |  |  |  |
| *Proceratophrys boiei* (Wied-Neuwied, 1824) | 6 |  | 6 |
| *Proceratophrys brauni* Kwet & Faivovich, 2001 |  | 1 | 1 |
| **Total** | 180 | 28 | 208 |

Figure S1 - Comparison of observed richness and estimated richness based on sample coverage completeness. The black points represent the observed richness values plotted against their corresponding estimated richness values, with a solid black line representing the relationship between these two variables.
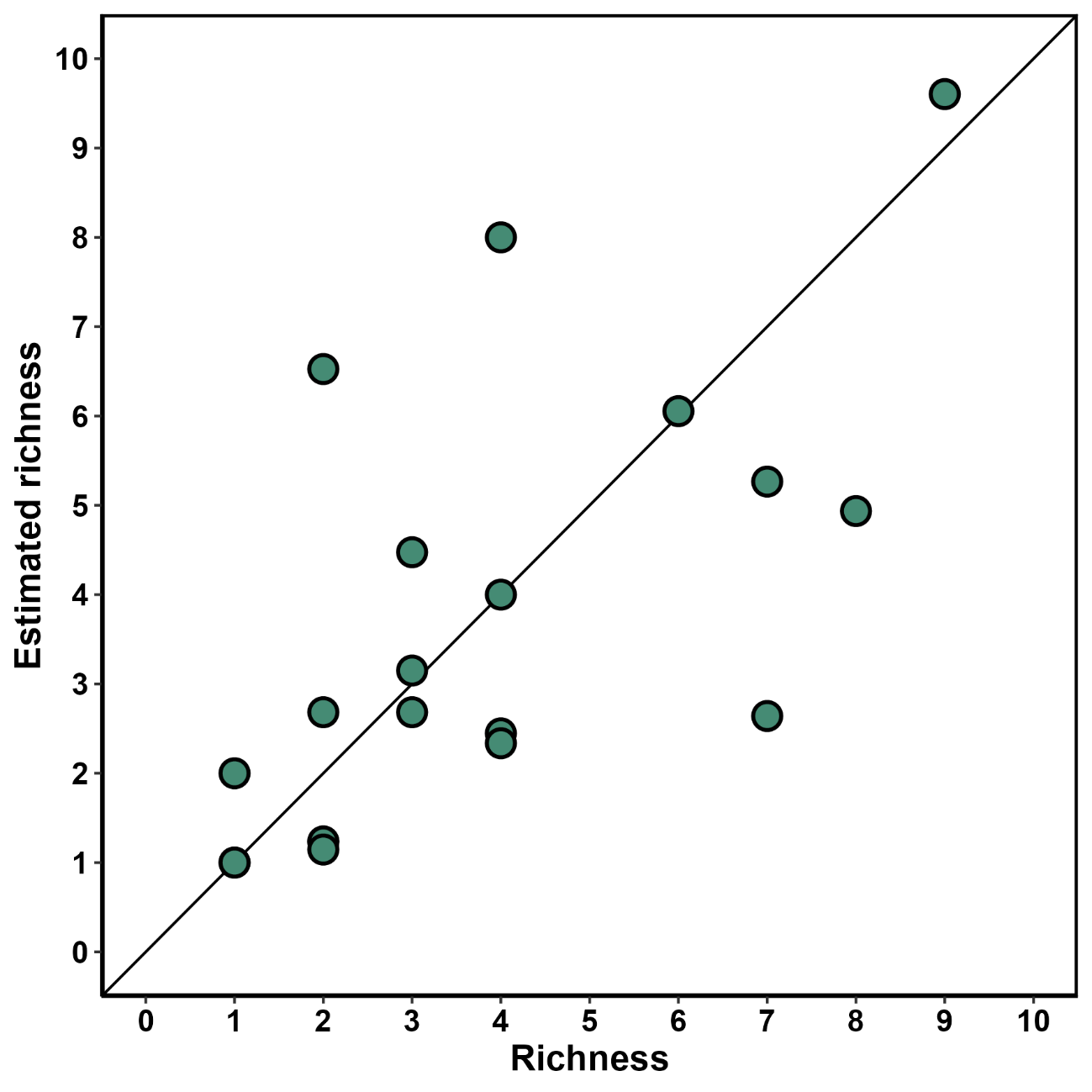


Table S3 - Results of GLMs using species richness and abundance as the dependent variables, temperature and net primary productivity as independent variables.

|  | **Variable** | **Estimate** | **S.E.** | **Z-value** | **P-value** |
| --- | --- | --- | --- | --- | --- |
| ***Species richness*** | |  |  |  |  |
| Model 1 | |  |  |  |  |
|  | Intercept | 2.2552 | 0.2531 | 8.908 | < 0.001* |
|  | Elevation | -0.0009 | 0.0002 | -3.696 | < 0.001* |
| Model 2 | |  |  |  |  |
|  | Intercept | 1.2101 | 0.1290 | 9.378 | < 0.001* |
|  | Temperature | 0.5232 | 0.1456 | 3.594 | < 0.001* |
|  | NPP | -0.1135 | 0.1595 | -0.711 | 0.476 |
| ***Species Abundance*** | |  |  |  |  |
| Model 1 | |  |  |  |  |
|  | Intercept | 4.0204 | 0.2619 | 15.347 | < 0.001* |
|  | Elevation | -0.0019 | 0.0002 | -7.121 | < 0.001* |
| Model 2 | |  |  |  |  |
|  | Intercept | 1.95095 | 0.1331 | 14.657 | < 0.001* |
|  | Temperature | 0.93638 | 0.1447 | 6.470 | < 0.001* |
|  | NPP | 0.05703 | 0.1759 | 0.324 | 0.746 |

The asterisks denote the significance level (p < 0.05).

Table S4- Results of GLMmv’s using species site composition as the dependent variable and area (low/high), temperature and net primary productivity as independent variables.

|  | **Variable** | **LR** | **P-value** |
| --- | --- | --- | --- |
| ***Species composition*** | |  |  |
| GLMmv 1 | |  |  |
|  | Intercept | 134.9 | 0.001* |
|  | Lowland | 108.7 | 0.001* |
| GLMmv 2 | |  |  |
|  | Intercept | 164.23 | 0.001* |
|  | Temperature | 105.33 | 0.001* |
|  | NPP | 26.84 | 0.164 |
| GLMmv 3 | |  |  |
|  | Intercept | 117.3 | 0.001* |
|  | Temperature | 111.9 | 0.001* |
| GLMmv 4 | |  |  |
|  | Intercept | 33.47 | 0.035* |
|  | NPP | 33.38 | 0.036* |

The asterisks denote the significance level (p < 0.05).

Table S5 - Set of GLMmv selection including pseudo R-squared, Akaike information criteria (AIC) and difference between best model AIC score and model AIC score (ΔAIC).

|  | **Species composition models** | **pseudo R-squared** | **AIC** | **ΔAIC** |
| --- | --- | --- | --- | --- |
| GLMmv 2 | ~ MAT + NPP | 0.36 | 612.74 | 21.16 |
| GLMmv 3 | ~ MAT | 0.30 | 591.58 | 0 |
| GLMmv 4 | ~ NPP | 0.09 | 670.06 | 78.48 |

Table S6 - Results of generalised linear regression models using beta diversity and its components for incidence-based and abundance-based index as the dependent variables and environmental distances as independent variables.

|  | Environmental distance | | | | |
| --- | --- | --- | --- | --- | --- |
|  | Elevation | | | | |
|  | Estimate | S.E. | z-value | P-value | Pseudo-R² |
| β_SOR_ | 0.0013 | 0.0001 | 8.799 | < 0.001 | 0.41 |
| β_SIM_ | 0.0016 | 0.0002 | 6.117 | < 0.001 | 0.06 |
| β_SNE_ | -0.0007 | 0.0001 | -4.003 | < 0.001 | 0.06 |
| β_BC_ | 0.0017 | 0.0001 | 10.429 | < 0.001 | 0.61 |
| β_BC.BAL_ | 0.0016 | 0.0002 | 6.401 | < 0.001 | 0.18 |
| β_BC.GRA_ | -0.0013 | 0.0002 | -4.841 | < 0.001 | 0.10 |
|  | Temperature | | | | |
|  | Estimate | S.E. | z-value | P-value | Pseudo-R² |
| β_SOR_ | 0.1174 | 0.0216 | 5.418 | < 0.001 | 0.60 |
| β_SIM_ | 0.0181 | 0.0078 | 2.311 | 0.02 | 0.23 |
| β_SNE_ | -0.1615 | 0.0719 | -2.244 | 0.02 | 0.06 |
| β_BC_ | 0.2846 | 0.0314 | 9.059 | < 0.001 | 0.59 |
| β_BC.BAL_ | 0.0359 | 0.0342 | 1.051 | 0.29 | 0.24 |
| β_BC.GRA_ | -0.2153 | 0.0677 | -3.180 | 0.001 | 0.08 |
|  | Productivity | | | | |
|  | Estimate | S.E. | z-value | P-value | Pseudo-R² |
| β_SOR_ | 0.0012 | 0.0002 | 5.856 | < 0.001 | 0 |
| β_SIM_ | 0.0008 | 0.0002 | 4.221 | < 0.001 | 0 |
| β_SNE_ | -0.0008 | 0.0001 | -4.466 | < 0.001 | 0.04 |
| β_BC_ | 0.0009 | 0.0001 | 5.542 | < 0.001 | 0.14 |
| β_BC.BAL_ | 0.0011 | 0.0002 | 4.869 | < 0.001 | 0 |
| β_BC.GRA_ | -0.0008 | 0.0001 | -4.211 | < 0.001 | 0.04 |

The asterisks denote the significance level (p < 0.05).
